# Work-related health information literacy: a factor analytic feasibility study of an adapted health information literacy screening tool (EHILS@work)

**DOI:** 10.3389/fpubh.2026.1974525

**Published:** 2026-10-22

**Authors:** Elke Ochsmann, Omeara Michael Collins

**Affiliations:** 1 Medical Faculty, Institute of Occupational Medicine and Public Health, Saarland University, Homburg, Germany; 2 Medical Faculty, Institute of Occupational Medicine, Prevention and Workplace Health Management, Lübeck University, Lübeck, Germany

**Keywords:** EFA (exploratory factor analysis), occupational health literacy, screening tools, work ability, workplace health promotion, work-related health information literacy

## Abstract

**Introduction:**

Health information literacy and health literacy increasingly intersect as important determinants of preventive behavior, health outcomes, and functioning at workplaces. Because organizations use a range of analog and digital communication channels and classical workplaces are replaced by hybrid work conditions, employees' ability to seek, evaluate, and apply work-related health information is gaining more relevance. However, these competencies remain insufficiently studied in workplace contexts. To address this gap, the Everyday Health Information Literacy Screening Tool (EHILS) was adapted for occupational use, resulting in the EHILS@work scale.

**Methods:**

A cross-sectional online survey was conducted among 1,108 employees of a public administration (*N* = 414, participation rate 37 %). The German EHILS was adapted through expert review to reflect work-related information contexts, and one exploratory item on intranet access was added (11 items in total). Descriptive analyses, exploratory factor analysis, reliability testing, and Spearman correlations with work ability and sociodemographic and workrole characteristics were performed.

**Results:**

Exploratory factor analysis (principal axis factoring with Promax rotation) supported a three-factor solution comprising “Awareness and Engagement”, “Assessment and Application”, and “Access and Understanding”. The three factors accounted for 38.3% of the variance, with the additional intranet item loading on the “Access and Understanding” factor. Compared with the original German EHILS scale (α = 0.50 −0.60), the adapted EHILS@work demonstrated improved, though still moderate, reliability for the three-factor solution (α = 0.658). Higher EHILS@work scores were positively associated with work ability, age, higher educational attainment, and leadership responsibilities.

**Discussion:**

The EHILS@work is a practical, time-efficient screening tool for assessing work-related health information literacy. It provides actionable insights into employees' competencies and perceived barriers within everyday organizational information environments. Broader validation, tailored information technology adaptions and longitudinal research are needed to examine its applicability across sectors and its role in shaping workplace health promotion.

## Introduction

Health information literacy and health literacy originated as distinct theoretical constructs, each grounded in different disciplinary traditions. Health information literacy is rooted in the information sciences and emphasizes the practices and capabilities required to search for, evaluate, and use information effectively. In contrast, health literacy grew out of health promotion and public health traditions, focusing on individuals' capacity to access, understand, and apply health information to maintain or improve health (1). Although their origins differ, contemporary scholarship has highlighted a substantial conceptual overlap between the two, with both fields increasingly recognized as critical determinants of preventive behavior, health outcomes, and healthcare costs (2–6). Moreover, health literacy has shown the potential to mitigate inequities within healthcare systems, serving as a valuable instrument for achieving health equity in alignment with the United Nations' “Sustainable Development Goal 3: Good Health and Well-Being” (7, 8).

Despite their rising prominence and potential, a recent WHO overview, which reports European results between 2019 and 2021, underscores that low health literacy—including deficits in health information literacy—remains a major public health challenge. Even in high-income regions such as Europe, approximately 46% of the population is estimated to have limited health literacy (a combination of categories “problematic” and “inadequate” health literacy) (9, 10). In view of earlier reports, e. g. Sorensen et al. [2015, (11)], which found limited health literacy in 47.6% of European respondents in 2011, it also underscores that for 10 years there was hardly any progress in general health literacy.

In occupational contexts, adequate health literacy among employees has gained increasing attention, as it is associated with work ability, employability, and workforce sustainability (12, 13) but also general health outcomes (14). Building on broader definitions of health literacy, occupational health literacy has been defined as the degree to which workers can obtain, communicate, process, and understand occupational health and safety information to make appropriate decisions regarding their work execution and workplace safety (15, 16). This construct encompasses the identification, retrieval and appraisal of relevant workplace information and the application of such information to support health-promoting behavior. Beyond individual skills, it also acknowledges that organizational structures may either facilitate or hinder access to trustworthy and comprehensible information or facilitate or hinder transferring this information into practice.

In many workplaces, information relevant to health, safety, or prevention is communicated through a combination of traditional channels (e. g., written guidance, team briefings, training sessions) and, in some cases, digital tools such as intranets or online materials. Because communication practices and information systems differ across organizations, assessments should reflect the actual environments in which employees encounter and use work-related health information, and hence need more focus on digital information sources. Nowadays, competencies for analog as well as digital information retrieval and interpretation shape how workers engage with the information structures embedded in contemporary organizations.

Despite recent developments, occupational and organizational health literacy—alongside related concepts such as workplace health information literacy—remain emergent fields. Theoretical frameworks and measurement approaches continue to evolve, and existing instruments are still undergoing conceptual refinement or address specific health outcomes (e. g. musculoskeletal health) (17–19). Importantly, to our knowledge, no brief screening tool currently exists that assesses everyday health information literacy in work-related information environments. Such tools might be of special interest for workplaces, as these are typically characterized by time constraints, diverse job roles, and often limited participation interest. A recent comparison of existing health literacy screening tools found significant differences in applicability and concluded that instrument selection must be guided by study objectives (20). Given the need for a concise measurement instrument suitable for large, diverse workforces, we identified the EHILS (German version) (21, 22) as a possible foundation. It was chosen because it assesses basic dimensions of health information literacy—motivation, confidence, and evaluation—without relying on disease-specific or clinical content. Its brevity makes it uniquely appropriate for occupational populations, and its adaptability has been demonstrated previously through successful modifications for assessing energy information literacy and attitudes toward digital health services in older adults (23, 24). However, as the original EHILS was developed for the general population outside the workplace context, its original form does not adequately capture the unique informational demands present in occupational settings.

To address this gap, we adapted the EHILS to reflect work-related health information literacy (EHILS@work) by rewording items to align with workplace information environments. This adaptation acknowledges that work-related health information is shaped by organizational communication channels, digital infrastructures, and employer-provided resources, all of which differ from general health information environments. Assessing employees' work-related health information literacy can thus inform the design of effective workplace health communication strategies and strengthen organizational health and safety culture (25).

Therefore, the aim of this study is to evaluate the psychometric properties of a workplace-adapted version of EHILS (EHILS@work), with particular attention to its factorial structure and measurement validity. Examining the scale within a large and diverse public administration workforce, contributes to research at the interface of occupational health, human factors, and organizational practice.

## Methods

### Participants and procedure

This study draws on data collected through an online employee survey conducted within a district administration in Northern Germany. The survey took place between March and April 2022 and was part of a broader evaluation of a newly developed stress-resource assessment tool designed for federal administrations (109 items). The 11 items of the workplace-adapted EHILS scale (EHILS@work) were added to the online survey, along with sociodemographic and health-related questions.

At the time of data collection, the administration employed 1,108 individuals across multiple departments, job functions, and locations, encompassing diverse levels of customer interaction, digital media exposure, and professional experience. All employees were invited to participate via internal communication channels, including the organizational intranet, email announcements, and written notifications distributed by management. Altogether 37% of the employees participated in the online survey. Participation was voluntary and anonymous, consequently differential participation of employees with varying levels of interest in health-related topics cannot be ruled out. Informed consent was obtained electronically from all respondents. Electronic informed consent was obtained from all participants. Ethical approval for the study was granted by the Institutional Ethics Committee of the University of Lübeck (AZ: 21-418).

The survey was administered using the SoSci Survey platform (www.soscisurvey.de), which complies with European data protection regulations (26). Data collection occurred over a four-week period, during which two reminders were issued to increase participation.

### EHILS@work

The adapted EHILS (EHILS@work) is based on the Everyday Health Information Literacy Screening Tool (EHIL) (22), respectively its German version (EHILS) (21). The original scale includes 10 items with reported internal consistencies ranging between α = 0.50 and α = 0.60 in German validation studies. Although modest, this reliability was considered acceptable for a short screening instrument for everyday health information literacy.

For the present study, the questionnaire was adapted to the workplace context through a structured, expert-driven process. An occupational health physician and a public health scientist independently reviewed and reworded all 10 original items to ensure relevance for work-related health information. This process focused on maintaining conceptual equivalence while aligning each item with the informational realities of contemporary organizations. For example, the general item “It is important to be informed about health issues” was revised to “It is important to be informed about work-related health issues”.

To reflect technology-mediated communication in modern workplaces, one additional exploratory item was introduced to assess employees' perceived accessibility of health-related information via the organizational intranet. This addition does not redefine the scale but acknowledges that many organizations now rely on digital platforms as one of several channels for disseminating occupational health information.

Consistent with the original EHILS, four negatively phrased items were reverse coded (e.g., “It is difficult to find work-related health information from the Internet”). The new intranet-related item was similarly formulated to preserve structural consistency. All items were rated on a five-point Likert scale ranging from 1 (“strongly disagree”) to 5 (“strongly agree”). The complete questionnaire is provided in the Supplementary Material.

### Sociodemographic variables, leadership role, and WAI (work ability index)

To assess concurrent validity of the EHILS@work scale, we conducted a cross-sectional analysis with some relevant sociodemographic (age, gender, schooling) and work-role-related variables (leadership tasks part of the work description). We also utilized a one item version of the Work Ability Index (WAI) assessment, commonly used in large-scale occupational studies (27, 28). Participants answered the question “Assume that your work ability at its best has a value of 10 points. How many points would you give your current work ability?” on a response scale between 0 = completely unable to work and 10 = work ability at its best. The one-item assessment of work ability has been shown to correlate with key outcomes such as sick leave and health-related quality of life (28), though it may be less sensitive in predicting disability pension or long-term sickness absence compared with the full WAI instrument (29, 30).

### Data analysis

Data processing included screening for missing values and removal of invalid cases. Only participants who completed all EHILS@work items were included in the analyses, diverging from the original German EHILS guidelines that allow up to two missing responses.

Descriptive statistics (means and standard deviations) were calculated and presented for individual items, the three emergent factors, and the overall EHILS@work scale.

Because Shapiro–Wilk tests indicated non-normal item distributions (all *p* < 0.001, not shown here), exploratory factor analysis (EFA) was conducted using principal axis factoring (PAF) as extraction method, as it is comparatively robust to skewness and kurtosis. Factor retention was guided by parallel analysis, scree plot inspection, and the Kaiser criterion. Because psychological constructs are expected to be correlated, we employed an oblique rotation (Promax) to obtain interpretable, simple structure while allowing for factor intercorrelations.

Items were retained if their primary loading was ≥0.30 with a cross-loading <0.275. Communalities were initialized using the squared multiple correlations and iterated to convergence. Analyses were replicated before and after excluding one item with low communality to assess structural robustness.

After establishing the factorial structure of the questionnaire EHILS@work instrument, we evaluated internal consistency using Cronbach's α and McDonald's ω, providing complementary reliability indices.

Concurrent validity was examined through Spearman rank correlations between EHILS@work total scale, and EHILS@work factors, sociodemographic and occupational variables (age, gender, educational background, leadership) and the WAI item. According to existing literature on EHILS (21, 22), we expected correlative associations between all sociodemographic variables and work ability.

All statistical analyses were performed using IBM SPSS Statistics (Version 29.0) and Jamovi (Version 2.4) with Jamovi supporting transparency and reproducibility of model outputs. (31–34).

## Results

### Participants

A total of 414 persons (37% of those invited) participated in the survey, of whom 326 (79%) completed all EHILS@work items and were included in the analysis. The sample reflected a rather typical demographic profile of regional public administrations in Germany, with women representing 73.8% of respondents (Table 1). Mean age was 43.6 years (± 11.4 years). Only 2.2% of participants reported lower secondary education, 35.1% finished intermediate secondary education, and 62.7 % reported a higher secondary educational background. The reported work ability ranged between 2 and 10, with a maximum at 8 points and a mean of 7.5 (SD = 1.4).

**Table 1:** Sociodemographic factors of the participants (*N* = 326 participants who answered all EHILS@work questions).

	*N*	%
Gender (*n* = 302)		
Male	84	26.3
Female	236	73.8
Age group (*n* = 296)		
18–30 years	48	16.2
31–40 years	60	20.3
41–50 years	85	28.7
51–65 years	103	34.8
Secondary education (*n* = 321)		
Lower	7	2.2
Intermediate	113	35.2
Higher	201	62.6
Leadership (*n* = 318)		
Senior management	2	0.6
Middle management	24	7.5
Project management	15	4.7
Employees	270	84.9
Trainees	7	2.2
WAI (*n* = 325)		
0–3	6	1.8
4–7	137	42.2
8–10	182	56.0

### Descriptive analysis of EHILS@work items

Descriptive analysis of the EHILS@work items can be found in Table 2. It revealed moderate variability in responses (e. g. 0.7 <SD <1.0). While most of the participants (84.4%) at least agree, that it is important to be informed about work-related health issues – the results point at some difficulties in assessing the reliability of work-related health information (approximately 25% report problems) and the transfer of work-related information to practice (approximately 35 % of respondents report problems).

**Table 2:** Adapted Questionnaire EHILS@work (11 items). (*N* = 326 participants who answered all EHILS@work questions).

EHILS@ work items	n (%)	1- strongly disagree	2	3	4	5- strongly agree	mean	SD
1) It is important to be informed about work-related health issues.	*n*	0	11	40	160	116	4.17	0.77
	%	0	3.4	12.2	48.9	35.5		
2) I know where to seek work-related health information.	*n*	4	62	66	146	49	3.53	1.00
	%	1.2	19.0	20.2	44.6	15.0		
3) I like to get work-related health information from a variety of sources.	*n*	3	28	90	155	51	3.68	0.87
	%	0.9	8.6	27.5	47.4	15.6		
4) It is difficult to find work-related health information from printed sources (magazines and books).^*^	*n*	33	97	114	69	14	2.80	1.01
	%	10.1	29.7	34.9	21.1	4.3		
5) It is difficult to find work-related health information from the Internet^*^.	*n*	63	152	63	46	3	2.31	0.97
	%	19.3	46.5	19.3	14.1	0.9		
6) It is easy to assess the reliability of work-related health information in printed sources (magazines and books).	*n*	10	65	131	102	19	3.17	0.91
	%	3.1	19.9	40.1	31.2	5.8		
7) It is easy to assess the reliability of work-related health information on the Internet.	*n*	6	76	123	100	22	3.17	0.92
	%	1.8	23.2	37.6	30.6	6.7		
8) Terms and sentences of work-related health information are often difficult to understand.^*^	*n*	63	134	77	48	5	2.38	1.01
	%	19.3	41.0	23.5	14.7	1.5		
9) I apply work-related health information to my own workplace and/or that of my colleagues and co-workers.	*n*	26	89	103	98	11	2.94	1.01
	%	8.0	27.2	31.5	30.0	3.4		
10) It is difficult to know who to believe in work-related health issues.^*^	*n*	48	88	135	51	5	2.62	0.97
	%	14.7	26.9	41.3	15.6	1.5		
11) It is difficult to find work-related health information in the company's/organization‘s intranet.^*^	*n*	40	98	108	72	8	2.72	1.02
	%	12.3	30.1	33.1	22.1	2.5		

^*^response scale inverted (mean values, SD, Var, IQR).

### Exploratory factor analysis (EFA)

Principal axis factoring (PAF) with Promax rotation was conducted after verifying factorability (Table 3). Measures of Sampling Adequacy ranged from 0.706 to 0.863, and the overall KMO value of 0.796 indicated good sampling adequacy. Bartlett's test of sphericity was significant (χ^2^(55) = 793, *p* < 0.001), confirming suitability for factor analysis. Parallel analysis and inspection of the scree plot supported a three-factor solution. In the initial model, factor loadings ranged from 0.301 to 0.803, and communalities ranged from 0.092 to 0.629. One item exhibited low communality and a primary loading just over 0.300 (Item 01). After removing this item, a second EFA yielded only slight improvements, while still supporting a three-factor structure, where one factor consisted of only two items. As no major improvements resulted from the exclusion of this factor and item 01 worked fair content-wise for factor building, item 01 remained in the final model in which three retained factors explained 38.3 % of variance.

**Table 3:** Factor loadings (EFA EHILS@work (11 items), principal axes factor analysis, Promax rotation).

EHILS@work items	Faktor			
	1	2	3	communality
EHILS@work_01			0.301	0.092
EHILS@work _02			0.496	0.444
EHILS@work _03			0.620	0.383
EHILS@work _06		0.803		0.614
EHILS@work _07		0.763		0.629
EHILS@work _09		0.322		0.171
EHILS@work _05^a^	0.680			0.511
EHILS@work _04^a^	0.628			0.371
EHILS@work _08^a^	0.465			0.287
EHILS@work _10^a^	0.556			0.454
EHILS@work_11^a^	0.559			0.259
**SS load**	1.842	1.507	0.863	
**% varicance explained**	16.75	13.70	7.84	
**Cumulative variance (%)**	16.7	30.4	38.3	

a: response scale inverted.

Based on item content, and in analogy to other authors, the three factors were re-sorted and interpreted as follows (see also Table 4).

- Factor 1*:* Awareness and engagement (Items 1–3)
- Factor 2*:* Assessment and application (Items 6, 7, 9)
- Factor 3*:* Access and understanding (Items 4, 5, 8, 10, 11)

**Table 4:** EHILS dimensions over time (2012–2026) depending on population and context.

Reports	Factor 1	Items	Factor 2	items	Factor 3	items
Niemelä et al. (22)^*^	motivation	1, 2, 3, 9	confidence	4, 5, 10	evaluation	6, 7
Mayer (35)^*^	motivation	1, 2, 3, 9	confidence	4, 7, 8, 10	(difficulty of) evaluation	–
Hirvonen et al. (1)^#^	awareness	1, 2, 3, 9	access	4, 5, 8, 10	assessment	6, 7
this study	awareness and engagement	1, 2, 3	access and understanding	4, 5, 8, 10, 11	assessment and application	6, 7, 9

^*^: factor 8 assessed as independent statement; ^**^: 2-factor solution possible (“motivation” and “confidence”) but not recommended because of internal consistency; ^#^: a Namibian sample produced a four factor structure. “Access” was subdivided in “understanding health information and recognizing relevant authorities” (items 8,10) and “finding information” (items 4,5).

A one-item solution, which was propagated in the original German version of the EHILS (21), was also calculated and used for comparisons.

To evaluate the robustness of the factor structure, also keeping in mind the low communality of one item, we additionally performed a principal component analysis (PCA). PCA also supported a 3-factor structure, though with relevant cross-loadings for item 10 and item 2. Total variance explained in PCA was 54.1%. We considered the similar component configuration as proof for the general stability of the identified latent structure. Given the conceptual aim of identifying latent constructs, we interpret and report the PAF solution as primary.

The original EHILS was developed around the dimensions motivation, confidence, and evaluation(22). Subsequent validation studies reported partially different structures, creating the dimensions awareness, access, and assessment proposed by Hirvonen et al. [(1); Table 4]. As the factor solution identified in the present study largely resembled this later structure, we used it as the basis for interpretation. Items 1–3 loaded on a factor labeled “Awareness and Engagement”, corresponding largely to the previously described awareness dimension. Items 6, 7, and 9 formed the factor “Assessment and Application” of Health Information, indicating that appraisal and practical use of health information are closely linked in the workplace context. Items 4, 5, 8, 10, and the newly introduced intranet item 11 loaded on a third factor labeled “Access and Understanding”, a reverse coded factor for perceived difficulties in locating, and understanding work-related health information. Overall, the factor structure was broadly consistent with previous EHILS research, suggesting that the core content domains of the instrument were retained following workplace adaptation, despite some differences in item allocation and factor labels.

### Reliability analysis

Internal consistency was evaluated using Cronbach's α and McDonald's ω (Table 5). For the original 1-factor (11-item) scale, as suggested by Mayer (2018), reliability was acceptable (α = 0.784; ω = 0.781), exceeding the widely cited criterion of ≥0.70 for research purposes (36), as well as exceeding the Cronbach's alphas found in the German EHILS version for the general population [0.50 – 0.60; (21)]. Exclusion of item 01 led to only slight increases in reliability (Cronbach's α = 0.789, McDonald's ω = 0.783), another hint at the stability of the identified 11-item solution.

**Table 5:** Reliability analysis (Cronbach's α, McDonald‘s ω; EHILS@work scale 1 factor (10 items) solution and 3 factor solution).

EHILS@work dimensions	Cronbachs α	McDonald's ω
EHILS@work scale (1 factor, 11 items)	0.784	0.781
EHILS@work scale (3 factors)	0.658	0.663
EHILS@work factor 1	0.504	0.539
EHILS@work factor 2	0.663	0.687
EHILS@work factor 3	0.727	0.728

Regarding the 3-factor structure, Cronbach's α as well as McDonalds ω decreased to 0.658 and 0.663, respectively, falling below the conventional threshold for adequate reliability. Although values around 0.60 may be tolerated in early stages of scale development or when factors contain few items (37, 38), they nonetheless indicate limited homogeneity and suggest that further item refinement may be necessary. The similarity between α and ω across both versions indicates that violations of tau-equivalence were minimal (39, 40). Overall, the results suggest that the original 11-item version demonstrates stronger internal consistency than the reduced three-factor structure, which might support the use of a one factor scale, which was already suggested by the authors of the German Version of EHILS. Nevertheless, all reliability analyses found alpha and omega reliability values above those of the original screening tool (or its German version), which suggests that context-specification – as in our case at workplaces - may improve the EHILS usage as a screening tool further on and therefore should be further elucidated before a final decision.

### Concurrent validity

We examined both the one-factor EHILS@work solution (total score) and the three-factor solution using Spearman correlation analyses (Table 6). The overall EHILS@work score was positively associated with work ability (WAI; *r* =0.266, *p* < 0.001). Similarly, all three dimensions of the EHILS@work showed significant positive correlations with work ability, namely “Awareness and Engagement” (*r* =0.246, *p* < 0.001), “Assessment and Application” (*r* =0.218, *p* < 0.001), and “Access and Understanding” (*r* =0.236, *p* < 0.001). These findings indicate that higher levels of work-related health information literacy are associated with better perceived work ability across all dimensions of the instrument.

**Table 6:** Spearman correlation between work ability and EHILS@work (1 factor and 3 factor solution).

			1 factor	3 factors		
Sociodemographic factors	Correlation coefficient	WAI	EHILS@work	EHILS@work _01	EHILS@work _02	EHILS@work _03
WAI	r		0.266^**^	0.246^**^	0.218^**^	0.236^**^
Age	r	0.087	0.187^**^	0.230^**^	0.160^**^	0.094
Gender	r	–.111^*^	0.043	0.045	0.032	0.001
Schooling	r	0.045	0.166^**^	0.117^*^	0.107	0.129^*^
Leadership	r	−0.065	0.175^**^	0.119^*^	0.156^**^	0.145^*^

^*^ *p* < 0, 05 (two-sided); ^**^ *p* < 0.001 (two-sided).

The three-factor solution further revealed several meaningful associations with sociodemographic and occupational variables. Both “Awareness and Engagement” (*r* 0.230, *p* < 0.001) and “Assessment and Application” (*r* 0.160, *p* < 0.001) increased with age, whereas no significant age association was observed for the “Access and Understanding” dimension. Educational attainment showed positive associations with “Awareness and Engagement” (*r* 0.117, *p* < 0.05) and “Access and Understanding” (*r* 0.129, *p* < 0.05). Furthermore, participants holding leadership responsibilities reported higher levels of “Awareness and Engagement” (*r* =0.119, *p* < 0.05), “Assessment and Application” (*r* =0.156, *p* < 0.001), and “Access and Understanding” (*r* =0.145, *p* < 0.05). No meaningful associations were observed for gender.

## Discussion

### Development of the EHILS@work

The development of the EHILS@work was guided by the increasing relevance of occupational health literacy in contemporary work environments [e.g. (12)] and the need to understand when and how employees seek, evaluate, comprehend, and apply work-related health information. Since the COVID-19 pandemic, work-related health literacy has gained distinct prominence in research and practice (41, 42), underscoring that a generic, ‘one-size-fits-all' approach, which treats health literacy as a general trait regardless of the setting, is often insufficient.

Evidence shows that both health literacy and health information literacy must be assessed within their specific contexts, as competencies are shaped by situational demands, information environments, and institutional structures (43, 44). Although workplace health literacy is influenced by organizational structures, leadership style, and information accessibility (12, 42), individual capacities remain central because they form the primary interface with organizational information systems. Accordingly, adapting the EHILS through context-specific rewording proved meaningful, acknowledging that employees encounter organization-specific communication landscapes distinct from private settings.

Occupational health literacy comprises knowledge-based competencies and responsibility-related components linked to work outcomes such as job performance and work ability (12). In organizational practice, such tools reveal communication barriers, access challenges, and structural factors shaping employees' navigation of work-related information. Short and concise screening tools like the EHILS@work reduce burden and survey fatigue and enable valid, large-scale assessments across heterogeneous roles—particularly relevant for cohorts spanning office, field, customer-facing, and mobile work. Brief, non-stigmatizing instruments also address concealment of low literacy due to shame or fear of judgement (45, 46) and help routinely identifying needs for targeted interventions, improving outcomes in workplace health (47). Brief instruments like the EHILS@work can also support periodic benchmarking, longitudinal monitoring, and informed strategy development.

### Descriptive analysis and derived EHILS@work cut-off values for practice

Our descriptive findings highlight that, compared with younger Finnish student populations, older employees place greater importance on being well informed about work-related health issues. This mirrors Eriksson-Backa et al. (23), suggesting that motivation to engage with health information may increase with accumulated work experience, exposure to occupational risks, and role-related responsibilities. In parallel, higher educational attainment and leadership roles were associated with higher EHILS@work scores in our cohort, consistent with well-described social gradients in health information engagement. Together, these patterns imply that both life-course factors (age/experience) and positional factors (education/leadership) shape how workers prioritize, seek, and appraise health information within organizational settings.

Based on the distribution of the sum scores of this study, we propose the following preliminary interpretive categories for the EHILS@work:

- ≤ 33 points: low work-related health information literacy
- 34–41 points: medium work-related health information literacy
- ≥ 42 points: high work-related health information literacy

These cut-offs—derived from the 25th and 75th percentiles—offer a pragmatic starting point for workplace screening and benchmarking across heterogeneous roles. Importantly, such bands can help organizations triage communication strategies (e.g., tailoring message complexity, selecting channels, calibrating support intensity) while monitoring equity-sensitive subgroups (e.g., non-leaders or employees with lower formal education). We emphasize that these thresholds are initial guidance, appropriate for local decision-making and longitudinal monitoring, and should be refined through validation in other sectors, job families, and information environments.

### EFA and internal consistency analyses

The exploratory factor analyses supported a three-factor solution—“Awareness and Engagement”, “Assessment and Application”, and “Access and Understanding”—which aligns with existing health literacy frameworks and EHILS-related findings (1, 5, 48–50). This configuration is also consistent with the view of occupational health literacy as a multi-layered construct shaped by individual, interpersonal, and organizational influences (12).

Context sensitivity was evident in item behavior. Item 9, which has previously aligned with motivational/awareness factors, loaded on assessment in our sample. We interpret this as a workplace-specific effect: applying health information at work is not only a function of motivation but is also bounded by feasibility, workflows, and organizational norms, which shape how information is evaluated and used on the job.

Although the one-factor solution yielded higher internal consistency, we retained the three-factor model because it offers greater diagnostic and implementation value (e.g., distinguishing motivational engagement from practical evaluation/use and from structural barriers). As expected, “Awareness and Engagement” showed lower reliability, reflecting the dynamic, multi-faceted nature of motivation; nevertheless, it remains central given its role in predicting health behavior change (51). Future iterations of the EHILS@work should enhance reliability in this domain while preserving conceptual breadth.

Maintaining conceptual depth is particularly relevant as many organizations struggle to provide accessible, comprehensible, and actionable health information – barriers that map closely to our third factor (42). Overall, internal consistency values were acceptable for an adapted, brief screening tool at an early developmental stage. Further validation across sectors with differing information practices and technologies will strengthen generalizability.

### Concurrent validity

The strong associations between EHILS@work (total and factors) with work ability underscore the practical relevance of work-related health information literacy for functional outcomes. These findings are consistent with evidence linking occupational health literacy with work ability (12) and mirrors earlier results in younger student cohorts (3). Beyond these direct relations, organizational research suggests that individual and organizational health literacy jointly influence well-being, with organizational health literacy potentially mediating these effects (42), a pathway that future studies on occupational health information literacy should test explicitly.

Age-related patterns—particularly for “Awareness and Engagement” (factor 1), and “Assessment and Application” (factor 2)—appear plausible, reflecting experience, exposure to occupational risks, and familiarity with work processes. These trends align with digital health literacy findings (23, 52) yet contrast with reported age-related declines in general health literacy (53, 54). The discrepancies highlight context-specificity and suggest that work-related literacy may develop positively with tenure and role-demands, even as general health literacy currently shows different age trajectories. This interpretation is consistent with the workplace focus of EHILS@work, which captures competencies developed and applied within occupational information environments. Consequently, accumulated work experience and repeated interaction with health-related information at work may offset or outweigh age-related disadvantages reported for more general health literacy constructs.

Finally, a social and hierarchy-based gradient remains evident. Lower education and non-leadership roles were associated with greater perceived barriers (55), indicating that structural advantages, such as higher formal education and positional authority, continue to shape how effectively employees navigate complex workplace information systems, irrespective of experience.

### Limitations

This cross-sectional design limits causal inference and precludes conclusions about the development or temporal stability of work-related health information literacy; longitudinal designs are needed to track change over time and in response to workplace interventions.

The sample derives from a single public administration, constraining generalizability. Private-sector, industrial, or more technologically intensive organizations may feature different communication practices and information demands. Voluntary participation may also introduce selection bias, potentially overrepresenting health-interested or more literate employees. Future studies should include more diverse sectors and use targeted outreach to engage less health-engaged subgroups.

Internal consistency was modest for some subscales, particularly motivation, indicating a need to refine items that may function differently in workplace versus general populations. Continued psychometric testing across occupational contexts is warranted.

Finally, reliance on self-report may not fully capture behavior. Integrating objective or behavioral indicators (e.g., intranet usage traces, observational methods, performance-based tasks) would provide a more comprehensive assessment of information seeking, comprehension, and application at work.

In sum, the EHILS@work shows promising usability as a practical, context-sensitive screening tool for work-related health information literacy. Its three dimensions—“Awareness and Engagement”, “Assessment and Application”, and “Access and Understanding” —offer more diagnostic value than a unidimensional approach and enable efficient assessment across heterogeneous roles. Given the rising importance of organizational health literacy and employees' ability to navigate work-related information systems due to the increasing changeability and complexity of work, EHILS@work can help identify individual competency gaps and structural communication barriers, informing targeted communication strategies and guiding improvements to organizational (including digital) infrastructures. This, in turn, can strengthen workplace health and safety culture and can support evidence-informed decisions at the interface of technology, organization, and worker well-being.

## Conclusions

EHILS@work is a promising brief screening instrument for assessing work-related health information literacy in occupational settings. The findings support its applicability as a workplace-adapted measure that captures employees' engagement with health information, their ability to assess and apply information, and perceived barriers to information access and understanding. Associations with work ability and selected sociodemographic, and occupational characteristics further support its relevance in workplace health research.

A particular advantage of EHILS@work is that it is not tied to a specific health topic, occupational sector, or organizational outcome. Instead, it captures a general competency that is relevant across different workplace health domains, communication channels, and intervention contexts. Consequently, the instrument can be used as a first-step screening tool to identify employee groups that may experience difficulties accessing, evaluating, understanding, or applying work-related health information before topic-specific interventions are developed.

## Data Availability

The datasets presented in this article are not readily available because of confidentiality requirements of the participating public administration. It can be made available on reasonable request. Requests to access the datasets should be directed to ls-arbeitsmedizin@uni-saarland.de.
