# Supplementary material for: Work-related health information literacy: a factor analytic feasibility study of an adapted health information literacy screening tool (EHILS@work)
Source: Front Public Health. 2026 Oct 22;14:1974525. doi: 10.3389/fpubh.2026.1974525 (PMC13736611; doi:10.3389/fpubh.2026.1974525)
Supplement: Supplementary file 1 [file Table_1.docx]

Supplement:

EHILS@work (workplace adapted EHILS questionnaire)

|  | | **1 Strongly disagree** |  |  |  | **5 Strongly agree** |
| --- | --- | --- | --- | --- | --- | --- |
| 01 | It is important to be informed about work-related health issues. | 🞏 | 🞏 | 🞏 | 🞏 | 🞏 |
| 02 | I know where to seek work-related health information. | 🞏 | 🞏 | 🞏 | 🞏 | 🞏 |
| 03 | I like to get work-related health information from a variety of sources. | 🞏 | 🞏 | 🞏 | 🞏 | 🞏 |
| 04 | It is difficult to find work-related health information from printed sources (magazines and books). ^a^ | 🞏 | 🞏 | 🞏 | 🞏 | 🞏 |
| 05 | It is difficult to find work-related health information from the Internet. ^a^ | 🞏 | 🞏 | 🞏 | 🞏 | 🞏 |
| 06 | It is easy to assess the reliability of work-related health information in printed sources (magazines and books). | 🞏 | 🞏 | 🞏 | 🞏 | 🞏 |
| 07 | It is easy to assess the reliability of work-related health information on the Internet. | 🞏 | 🞏 | 🞏 | 🞏 | 🞏 |
| 08 | Terms and sentences of work-related health information are often difficult to understand. ^a^ | 🞏 | 🞏 | 🞏 | 🞏 | 🞏 |
| 09 | I apply work-related health information to my own workplace and/or that of my colleagues or coworkers. | 🞏 | 🞏 | 🞏 | 🞏 | 🞏 |
| 10 | It is difficult to know who to believe in work-related health issues. ^a^ | 🞏 | 🞏 | 🞏 | 🞏 | 🞏 |
| 11 | It is difficult to find work-related health information from the Company’s/ Organization’s Intranet. ^a^ | 🞏 | 🞏 | 🞏 | 🞏 | 🞏 |

^a^ inverted variable
